# Personalised care, youth mental health, and digital technology: A value sensitive design perspective and framework

**DOI:** 10.1007/s10676-025-09866-x

**Published:** 2025-10-22

**Authors:** Adam Poulsen, Ian B Hickie, Min K Chong, Haley M LaMonica, Ashlee Turner, Frank Iorfino

**Affiliations:** Brain and Mind Centre, The University of Sydney, Sydney, Australia

**Keywords:** Ethical technology assessment, DMHT, Person-centred care, VSD

## Abstract

Digital health is typically driven, in part, by the principle of personalised care. However, the underlying values and associated ethical design considerations at the intersection of personalised care, youth mental health, and digital technology are underexplored. Through a value sensitive design lens, this work aims to contribute a prototype conceptual framework for the ethical design and evaluation of personalised youth digital mental health technology, which comprises three values–personalisation, empowerment, and autonomy–and 15 design norms as fundamental yet non-exhaustive ethical criteria. Furthermore, it provides illustrative applications of the framework by applying it to (1) the proactive design of two exemplary digital mental health technologies to draw out emerging ethical considerations and (2) the retrospective evaluation of three existing technologies to assess whether they are designed to support personalisation, empowerment, and autonomy. This work creates an understanding of personalised care and related values in this socio-technical context, with key design recommendations going forward for youth digital mental health research, practice, and associated policy.

**Supplementary Information:**

The online version contains supplementary material available at 10.1007/s10676-025-09866-x.

## Introduction

Digital health technologies (DHTs) are expected to support personalised care, owing to substantial data collection and processing capabilities and context-driven adaptability. DHTs framed as providing personalised care include robotics (Umbrello et al., 2021), artificial intelligence (Fosch-Villaronga et al., 2021), persuasive technology (Henkemans et al., 2015), mobile health apps (Daud et al., 2023), smart cities technologies (Zhou et al., 2023), machine learning (Nguyen & Poo, 2017), and various digital mental health technologies (DMHTs) (Burr & Morley, 2019; O’Toole, 2021). Personalised care principles play a role in developing DHTs in a variety of areas, including health promotion (Henkemans et al., 2015), direct caregiving activities (Fosch-Villaronga et al., 2019; Tan et al., 2021), diagnosis and monitoring (Peeters et al., 2021), self-management (Daud et al., 2023), promoting social inclusion and participation (Zhou et al., 2023), and health data collection (Nguyen & Poo, 2017).

Frequently, the DHT literature ambiguously refers to the ‘value of personalised care’ (Stafford et al., 2020; Ventura et al., 2023). Research suggests that personalised DHTs increase and improve utility, effectiveness, and efficacy factors (e.g., data processing, progress tracking, medication optimisation, self-management behaviours, and tailored health information) and, to a lesser extent, value-add factors (e.g., patient empowerment and user experiences) (Hickie et al., 2019a, 2019b; Madanian et al., 2023; Slevin et al., 2019; Vervoort et al., 2022). Yet, the substantial focus on evaluating DHTs for functionality and impact risks obscuring ethical or value-related considerations, including those concerning personalised care, given that they are often forgotten, understudied, or poorly regulated (Burr & Morley, 2019; Iqbal & Biller-Andorno, 2022; Unsworth et al., 2022). In youth mental health specifically, there remains a persisting need to identify the characteristics and preferences of youth and develop personalised DMHTs to improve effectiveness and value-add for this target audience (August & Gewirtz, 2019). At present, the underlying values and associated ethical design considerations at the intersection of personalised care, youth mental health, and digital technology are underexplored. There is a clear need for a framework to address ethical challenges associated with designing personalised DMHTs for young people, guiding proactive ethical technology assessment and retrospective evaluation of existing technologies going forward (Wies et al., 2021).

This work addresses this gap, aiming to make several contributions. First, a value sensitive design perspective of personalised care generally and youth digital mental health specifically. Second, a prototype conceptual framework for the ethical design and evaluation of personalised youth DMHT (hereafter referred to as the Framework) by conceptualising personalised care using various literature to draw out key underlying values and design norms. The Framework comprises a set of three key values–personalisation, empowerment, and autonomy–and 15 design norms (5 norms per value) as fundamental yet non-exhaustive ethical criteria to personalised care in this context. Third, two illustrative applications of the Framework to (1) the proactive ethical design of two DMHTs to draw out emerging ethical considerations and (2) the retrospective evaluation of three existing DMHTs to assess whether they support personalisation, empowerment, and autonomy.

## Value sensitive design

Value sensitive design (VSD) is a technology design methodology supporting the design of systems sensitive to a particular socio-technical context of stakeholders, values, and technologies (Friedman & Hendry, 2019). It maintains that technology is not value-neutral (Friedman, 1997), and stakeholders should be directly involved in the design process (Friedman et al., 2013). Following VSD theory, values are described as ‘what is important to people in their lives, with a focus on ethics and morality’ (Friedman & Hendry, 2019). VSD realises values, embeds them into design, and evaluates technologies using a tripartite methodology comprised of three iterative investigations (Friedman & Hendry, 2019). First, conceptual investigations aim to identify key stakeholders, conceptualise their values in relation to the technology and its application environment, and begin to translate values into conceptual design norms and requirements. Second, empirical investigations seek to empirically deepen the contextualisation of values, norms, and design requirements and reveal how stakeholders apprehend values in the observable context, competing values, and value trade-offs. Third, technical investigations aim to enrich understanding of the relationship between values and technical properties, as well as practically realise (and evaluate) design artefacts that support the values and examine how they may hinder values (Friedman et al., 2013).

Applying VSD, this work presents a conceptual investigation. It focuses on *value conceptualisation* to define, describe, analyse, and draw out the meaning and applicability of values, and then later illustrative *specification* to generate specific design norms and requirements for those values (van de Poel, 2013). Previous VSD research has similarly examined and explicated criteria for informed consent in online interactions to design web browser cookie mechanisms (Friedman et al., 2013). Recent literature has likewise applied VSD as a lens for examining various technologies, including databases (Jaljolie et al., 2023), blockchain (Ietto et al., 2023), and self-sovereign identity solutions (Ishmaev et al., 2023).

To develop the early-stage conceptual Framework and later apply it in practice, the activity of specification is required. The specification process is informed by context- and domain-specific knowledge and follows two steps: 1) the translation of values into norms (i.e., prescriptions for, and restrictions on, designed artefacts, including properties, attributes, capabilities, objectives, goals, and constraints), and 2) the translation of norms into specific design requirements (van de Poel, 2013). Specification produces a values hierarchy consisting of values, norms, and design requirements for any given design artefact.

In the same way that Umbrello and van de Poel (2021) developed a novel VSD approach based on AI for Social Good principles, this work prototypes the Framework based on a conceptual investigation of personalised youth DMHT. Umbrello and van de Poel (2021) identified values associated with the AI for Social Good principles and mapped them onto norms to elicit technical design requirements. Likewise, the Framework prototyped here is mapped to a values hierarchy. However, given the context-specificity associated with different DMHT solutions, the Framework only initially populates the values and norms components of the values hierarchy. Yet, sufficient contextualisation, i.e., drawing on the context of youth digital mental health, appropriately guides this conceptual investigation, enabling the formulation of the norms applying van de Poel’s (2013) criteria for the adequacy of a certain value-to-norm translation. These criteria require that a norm is an appropriate response to the value and that it is sufficient to respond to or engage with the value. In the later sections, the Framework is proactively applied to design two exemplary DMHTs, eliciting specific illustrative design requirements and thus completing the values hierarchy for those specific DMHTs. In this instance, van de Poel’s (2013) criteria for norm-to-design requirement translation are followed, ensuring that the requirement meets the scope of applicability of the norm, the goals or aims strived for, and actions or means to achieve these aims. Additionally, in a subsequent section, the Framework is retrospectively applied to three existing DMHTs to evaluate whether they are designed to support personalisation, empowerment, and autonomy.

## Personalised care and digital mental health technology design

In healthcare broadly, personalised care draws focus to care that adheres to a data-driven and tailored approach, using data to develop personalised care pathways for individuals based on their biomarkers, prognosis, demographics, and medical history (Mitchell et al., 2023; Van Staden et al., 2023). Personalised care emphasises precision and incorporates evidence-based processes, and without a range of measurements, from biological to socioeconomic factors, personalised care falls short (Hickie et al., 2019a). It is also closely linked to advancing proactive self-care and promoting the values of empowerment and autonomy (Entwistle et al., 2022; Johnson et al., 2023). In mental healthcare, personalisation in care describes ‘the notion that the assessment of, and the sequence of interventions for, mental disorders are tailored to the individual, and their changing needs over time’ (Iorfino et al., 2019). It is directly linked to data-driven, real-time, measurement-based approaches to mental healthcare, drawing on rich datasets capturing a person’s, for instance, physical comorbidities, individual preferences, personal history, cognitive functioning, environment, and lifestyle (Hickie et al., 2019a; Mayer et al., 2023).

**Fig. 1:**
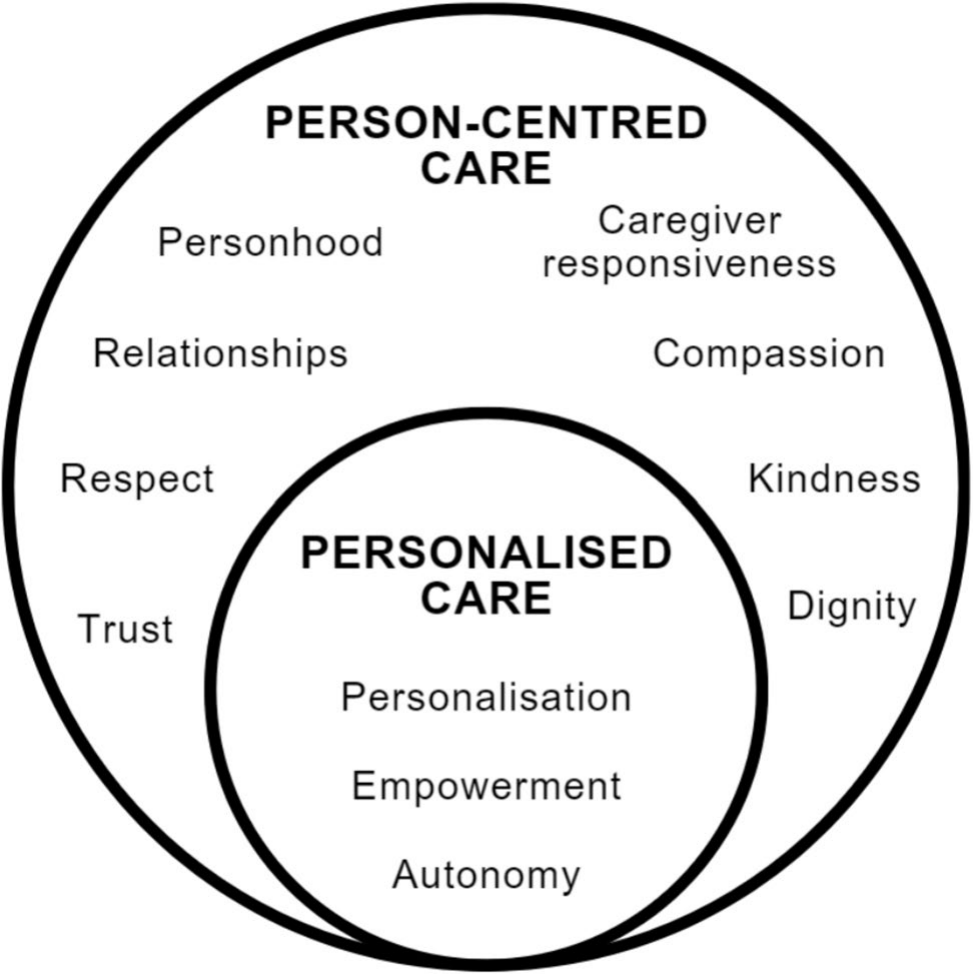
Values implicated in person-centred care and personalised care

It is important to note that personalised and person-centred care are often used synonymously in the literature (Santana et al., 2018). Here, personalised care is differentiated from (or a subset of) person-centredness in care, which describes a holistic view of the individual in treatment (Mezzich et al., 2011). In other words, personalised care is an approach (Hickie et al., 2019a) to principled person-centred care (Gondek et al., 2017). As a recurring theme, the reciprocal relationship between the values of personalisation, autonomy, and empowerment is regularly cited in the health services literature (Carr, 2010; Cesuroglu et al., 2016; Lewis & Sanderson, 2011; Rummery et al., 2023). Personalised care primarily concerns a narrow range of values emerging from the focus on data-driven intervention and tailored care pathways (i.e., personalisation, empowerment, and autonomy) compared to the more holistic overarching concept of person-centred care. As a part of person-centred care, personalised care also implicates the broader set of values nonetheless, including personhood, hope, ongoing collaboration, equal partnerships, caregiver responsiveness, respect, dignity, trust, compassion, and kindness (Entwistle et al., 2022; Mayer et al., 2023), albeit to a lesser extent (see Fig. 1).

In mental healthcare, understanding a client’s experiences, values, goals, needs, etc. has always been at arm’s length (Moggia et al., 2024). Seeking to develop understanding is typically done through a process of clinician observation or a client communicating their needs to a clinician. However, this process is dependent on and limited by specific contexts, time, communication skills, etc. (Moggia et al., 2024). In this way, first understanding a client to then subsequently improve personalised care is limited by a clinician’s lens, presenting a risk of the individual getting lost in the clinician’s interpretation. In the digital age, the proliferation of DMHTs has enabled a shift in power dynamics, presenting an opportunity to benefit access to care, greater personalisation, empowerment, and autonomy among clients, and an improved understanding of lived experiences. However, if the design of DMHTs is limited to the clinician’s lens without a broader view of the socio-technical ecosystem, consideration for the client’s voice, and understanding of the underlying values, the same drawbacks will be exacerbated, risking failure in delivering personalised care. This particularly puts vulnerable groups at risk, such as young people. Thus, going forward, it is crucial to develop an understanding of personalised care considering the wider socio-technical context, stakeholders’ perspectives, and fundamental values.

## Personalisation plus: empowerment and autonomy

Personalisation, empowerment, and autonomy are salient values in the context of personalised youth digital mental health. Altogether, these values form the foundation of the prototype conceptual Framework. However, following VSD, any list of values is non-exhaustive (Friedman & Hendry, 2019), so the Framework serves to provide fundamental yet non-exhaustive ethical criteria; other values can and should be accounted for as they emerge. The following sections further elaborate on these values based on the VSD, DHT, DMHT, and youth mental health literature. This section explicitly labels emerging norms using parentheses to highlight the link between value conceptualisation and specification that form the Framework.

### Value of personalisation

Following the VSD literature, personalisation is an ethical value of import in technology design (Bednar & Spiekermann, 2022; Bednar & Spiekermann-Hoff, 2020; Bozdag, 2013). That is, personalisation is desirable and raises ethical considerations, thus meeting the defined criteria of a value in VSD theory as ‘what is important to people in their lives, with a focus on ethics and morality’ (Friedman & Hendry, 2019). Bednar and Spiekermann (2024) describe the value of personalisation as the adaptation to ‘the user’s skills (e.g., biker-friendly roads, age-appropriateness) and/or the user’s preferences (e.g., language)’. Sharon (2023) argues that personalisation, along with the value of optimisation, is a core value of technology inherently linked to the emergence of digitisation and the substantial increase in the volume of data (*Norm 1: Collect and integrate large datasets*). Tensions between personalisation and other values exist. For instance, excessive content personalisation to increase relevancy to users risks creating information echo chambers around users and thus bias, resulting in negative implications for other values, including autonomy, transparency, objectivity, serendipity, privacy, and trust (Bozdag, 2013; Custers et al., 2018; Giovanola & Tiribelli, 2023). To alleviate these tensions, research shows that overt personalisation of digital technologies (facilitated by increased transparency and user control over personalisation features such as frequency of messages) decreases privacy concerns and increases user autonomy, confidence, engagement, and acceptance (Garrido et al., 2022; Jaljolie et al., 2023) (*Norm 2: Ensure overt personalisation*). 

Personalisation of DHTs is often identified as a means for *moving towards personalised care* in DHT research (Drake, 2014; van Hees et al., 2023; Vervoort et al., 2022) and is positively linked to other values, such as empowerment, self-management, responsibility, autonomy, solidarity, and safety (Asbjørnsen et al., 2020; van Hees et al., 2023). Yet, personalisation has intrinsic value. In tailoring health delivery, information, interventions, and DHTs to the distinct characteristics, contexts, goals, and preferences of the individual, personalisation respects and affirms one’s identity and lived experience as integral to care (Carter et al., 2018; van Dooren et al., 2019; Rummery, 2011). In other words, even in instances where personalisation does not lead to better health outcomes, it is ethically and socially valuable as it affirms the individual as an active participant in their care. For example, adapting the tone, language, and cultural references of a DHT to accommodate a user’s personal identity is valuable in itself, regardless of measurable clinical impact. Essential to advancing personalisation, DHTs should collect and integrate data and utilise large datasets (Cancela et al., 2021) (*Norm 1*) and follow an evidence-based approach (Hickie et al., 2019a) (*Norm 3: Follow an evidence-based approach*). As a part of the dynamic care milieu, the value of personalisation as it relates to DHT is fluid and requires balancing with (1) other interconnected values, including cost-efficacy and data security (van Hees et al., 2023) and (2) too much versus too little personalisation to fairly accommodate the diverse needs of those outside the typical user profile and ensuring scalability for the typical user profile (Burr & Morley, 2019) (*Norm 4: Account for user characteristics*).

There is limited literature on DMHTs broadly and the value of personalisation. Some research suggests that personalisation exists in the relationship between the user and the system, playing a role in enhancing personalised interactions to improve outcomes (Burr & Morley, 2019; O’Toole, 2021). Additionally, personalisation is typically framed as essential to supporting the optimisation of individual outcomes by integrating various forms of data collection, making comparisons to large datasets, and making clinical recommendations that align with individuals’ unique characteristics, needs, and circumstances (Moggia et al., 2024) (*Norm 1; Norm 4*).

In youth digital mental health contexts, personalisation is important as it ensures ‘fit’, i.e., the tailoring of care strategies to young people’s needs and expectations. A study by van Dooren et al. (2019) on gamified DMHTs reported that greater personalisation illustrates to young people a clearer link between their real-world experience and the potential benefits of mental healthcare, thus helping to manage expectations. For instance, incorporating highly personalised goals, tasks, and content into DMHTs that are tailored to a young person’s interests better communicates to users how to achieve certain goals in specific situations and illustrates goal achievement and failure patterns (van Dooren et al., 2019). In another study, Kornfield et al. (2023) highlight that personalisation ensures that DMHT-supported care programs accommodate each individual’s needs, preferences, and contexts (e.g., availability and psychological states). The study employed mobile phone messaging to send information to young people who reported wanting greater personalisation, specifically the capability to elect preferences for the volume, timing, and content of messages received (*Norm 4*). Furthermore, the authors found that young people using DMHTs valued a dynamic and individualised user experience (UX) and warned of the need to balance potentially burdening requests for user feedback that help to personalise a DMHT and an uninterrupted UX (Kornfield et al., 2023) (*Norm 5: Balance feedback requests for personalisation with an uninterrupted UX*). The added value of personalisation includes its role in enabling support for those who are indifferent to standardised care, accounting for a diversity of characteristics represented in the youth mental health (e.g., the broad age range), accommodating the needs of marginalised and hard-to-reach youth (e.g., homeless youth), and accounting for different beliefs and expectations and alleviating culture-based stigma that impacts help-seeking behaviour among young people (e.g., shame) when developed with cultural sensitivity in mind (Cillin, 2022) (*Norm 4*).

Following the activity of specification, in which values are translated into norms and design requirements (van de Poel, 2013), Fig. 2 maps the emerging norms in the above conceptualisation of the value of personalisation.

**Fig. 2:**
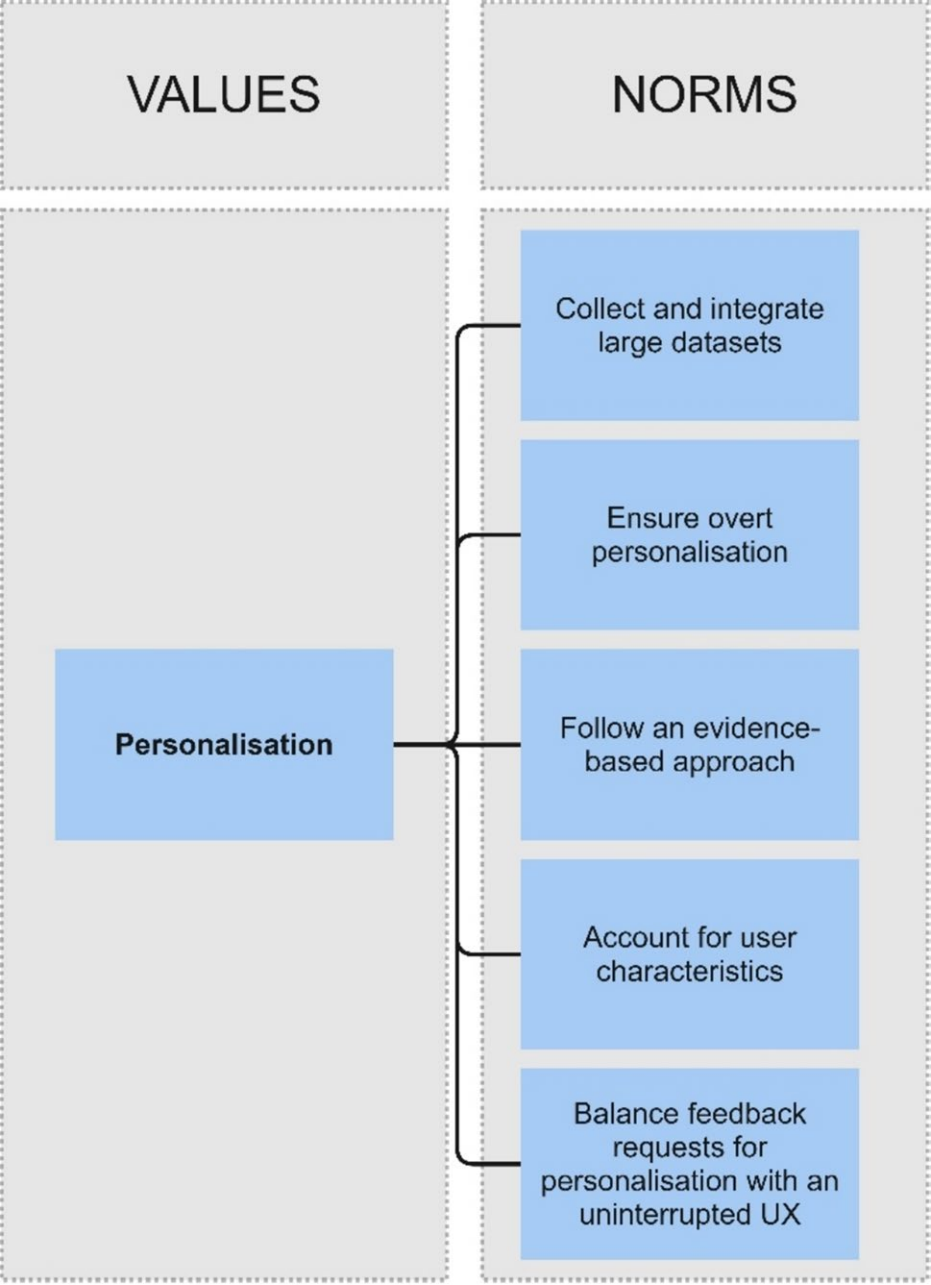
Value of personalisation component of the Framework

### Value of empowerment

‘Empowerment is a term that is used both loosely and inconsistently’ (Burr & Morley, 2019). The potentially misleading language of empowerment risks overselling the degree of control over health and health outcomes that DHT users can expect (Nickel et al., 2023) and necessitates creating a richer understanding of the value.

In the VSD literature, empowerment is reported as among the ‘crucial ethical virtues for human development’, alongside cooperation, curiosity, creativity, and reflectivity (Cenci & Cawthorne, 2020). Deng et al. (2016) directly contrast empowerment with marginalisation and note four dimensions of empowerment expression: meaning, competence, self-determination, and impact. Empowerment is linked to the value of autonomy (e.g., when practices, standards, and policies support autonomy, one may feel empowered) (Deng et al., 2016). Some studies describe empowerment as an essential step towards different forms of bottom-up participation (e.g., social participation) and away from top-down control (Ahmadi et al., 2022; Helbing et al., 2021). Realising empowerment depends on balancing individual factors within the average person’s control and outside factors. For instance, the substantial volume of information (and potentially incorrect information) available in the digital age does not automatically guarantee the empowerment of individual recipients as it requires (1) assurances by sources for accurate, reliable, and relevant information and (2) individual skills to adequately use the information and thus empower oneself (Pols & Spahn, 2015).

DHTs are frequently linked to advancing empowerment by enabling people to, for instance, ‘take care into their own hands, improve monitoring and rehabilitation, and expand access to care’ (Vervoort et al., 2022). Empowerment, among other things (i.e., self-management and personalisation), is a driving factor in adopting DHTs (Madanian et al., 2023). DHT research shows that empowerment is linked to improved patient participation in clinical decision-making, patient-clinician relationship, control, health education, and reduced technology-related frustration (Madanian et al., 2023). To empower users, DHTs intend to enable accurate self-management, support diagnosis, and provide a means for improved patient-clinician communication and shared decision-making (Slevin et al., 2019) (*Norm 6: Support health–self-management and diagnosis; Norm 7: Support reciprocity–shared decision-making and patient-clinician communication*). For instance, one may feel empowered to participate in shared decision-making with health professionals owed to the accuracy of self-managed health data facilitated by DHTs rather than relying on recall (Slevin et al., 2019).

Yet, typically, DHTs are best fit to reach and empower people with high technological and health literacy, leaving others behind and disempowered (Nickel et al., 2023). Addressing empowerment in the design, development, and implementation of DHTs requires addressing broader social factors impacting access and any potential mismatches between developer assumptions about users and actual user expectations, values, competencies, and backgrounds (Nickel et al., 2023) (*Norm 8: Account for wider socioeconomic and social factors affecting use and access*). Empowered individuals in care are not necessarily only those who are technologically empowered to become highly active, making self-initiators of decisions related to their health, but also those making small improvements in their capability to access and use health resources and services (*Norm 9: Accommodate different levels of empowerment*). For instance, empowered persons also include those who feel empowered to agree to treatments recommended by health professionals and those empowered to engage in shared decision-making (Nickel et al., 2023). In this way, empowerment supported by DHTs is linked to the values of justice, respect, autonomy, and individual responsibility (Nickel et al., 2023) (*Norm 10: Support the self–responsibility*).

Burr and Morley’s (2019) account of empowerment relating to DMHTs is highly relevant here. Empowerment is more than patient self-monitoring, enabling self-reflection, providing information directly to individuals, and distributing tasks to patients (*Norm 10: Support the self–self-reflection and information and task distribution*). It requires an adequate account for wider socioeconomic factors that, if otherwise left unmitigated, risk negatively affecting an individual’s freedom to decide whether to engage with DMHTs (*Norm 8*). Yet, typically, the framing of empowerment in mental healthcare ‘tends to ignore the fact that there are many factors that moderate an individual’s ability and motivation to even engage with this active process of self-reflection’ (Burr & Morley, 2019). Empowerment and autonomy are linked. By definition, empowerment enables autonomy (Burr & Morley, 2019). In mental healthcare, a major value dependency between empowerment and autonomy emerges: certain psychiatric disorders impact decisional capacity and thus autonomy, restricting the ability to realise empowerment (Burr & Morley, 2019).

A study by Maathuis et al. (2020) highlights the saliency of empowerment in VSD studies on DMHTs, suggesting that it is among the values at stake for the future users of digital technologies. The authors warn that in striving to advance empowerment, a tension between empowerment and universal usability emerges. DMHT designers might seek to promote access to content or self-reporting assessments by using overly simplistic language, risking infantilising users with ‘childish language’ and thus disempowering users (Maathuis et al., 2020). Another clear value conflict emerges in this space: empowerment versus efficiency. Enabling users to decide who to share their health data with for the sake of patient empowerment might reduce the efficiency of health professional practice in the delivery of mental healthcare (and potentially the efficiency of policymakers and insurers implicated in this context). The authors resolved this tension by dividing data into two domains: core data that must be shared relating to more universal facets (e.g., safety, living situation, finances) and add-on data that clients can decide whether to share relating to more individual facets (e.g., social contacts, leisure, lifestyle). In effect, the authors draw a compromise between the values held by the two parties, ultimately disempowering clients for the sake of professional efficiency and vice versa (Maathuis et al., 2020). Empowerment is closely positively linked with ownership (i.e., owning one’s own health), autonomy, independence, control, and self-drive, as well as negatively related to increased responsibility or obligation and imposed control (Maathuis et al., 2020) (*Norm 10: Support the self–responsibility and active involvement*). At risk is the overreliance on clients taking their health measurements and managing their health data among government and health insurers, imposing an obligation on clients and ultimately disempowering individuals in an effort to empower them (Maathuis et al., 2020).

Although it has been implied until this point, it should be noted that the value of empowerment is regularly considered interchangeably both as a means and an end. As an end, the value of empowerment is commonly described in youth digital mental health contexts as an intrinsic sense or feeling resulting from one’s increased active involvement in their care and shared decision-making through, for instance, digitally-mediated self-monitoring, self-management of symptoms, and help-seeking behaviours (Kerber et al., 2023; Lehtimaki et al., 2021; Peck et al., 2020; Wies et al., 2021) (*Norm 10: Support the self–active involvement*). Much of the literature focuses on achieving empowerment through greater health literacy and suggests that empowerment depends on the accuracy of available information (Chapman et al., 2017).

As a means, empowerment is often cited as instrumentally valuable in increasing help and information seeking, quality of life, responsibility, and capability-building, as well as in reducing stigma. Huggett et al. (2017) note that empowerment is a motivator to, for instance, seek out help or information, and digital technology is framed as an enabler of that motivation. Likewise, Chapman et al. (2017) suggest that greater empowerment results in improved capability-building, such as improving one’s skill to communicate personal values in decision-making (*Norm 10: Support the self–capability-building*). An increased sense of empowerment is also associated with improved quality of life and increased responsibility (Kerber et al., 2023; Wies et al., 2021). Some studies draw a connection between increased empowerment and reduced stigma. That is, empowering young people to monitor and understand their mental health potentially creates a knock-on effect, reducing stigma about one’s mental health and, as a broader collective outcome, about mental health on a societal level (Lehtimaki et al., 2021; Wies et al., 2021). As either a means or an end, it is clear that empowerment is valuable, playing a key role in personalised youth digital mental health contexts (Kerber et al., 2023; Lehtimaki et al., 2021; Peck et al., 2020; Wies et al., 2021).

**Fig. 3:**
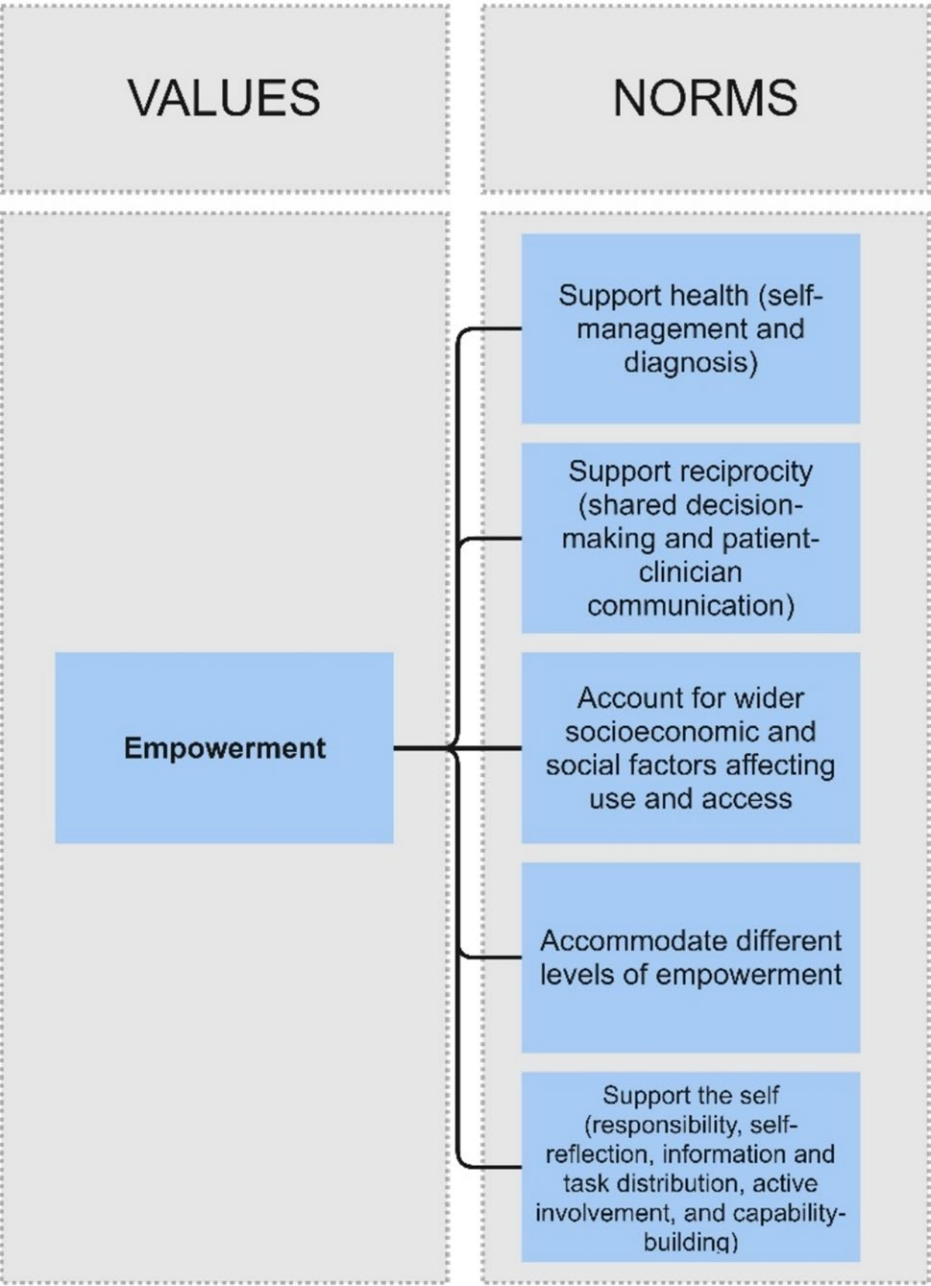
Value of empowerment component of the Framework

Figure 3 presents a schema of the norms relating to empowerment as described above.

### Value of autonomy

Before the value of autonomy is examined, it must be disentangled from the value of choice, as the two terms are often used interchangeably. As a principle of biomedical ethics, autonomy is ‘the capacity of the person to deliberate or act on the basis of one’s own desires, that is the ability to act freely in accordance with a self-chosen plan’ (Wies et al., 2021). Furthermore, limitations to capacity refer to possible impairments (e.g., visual impairment or intellectual disability), affecting one’s capacity to interact with others or systems. Capacity and possible limitations to capacity are key components of autonomy (*Norm 11: Address capacity or possible limitations to capacity*). Beauchamp and Childress (2013) point out that autonomy is ‘self-rule that is free from both controlling interference by others and from limitations, such as inadequate understanding, that prevent meaningful choice’ (*Norm 12: Support self-directedness–choice*). From another’s perspective, respect for someone else's autonomy is ‘the obligation to respect the decision making capacities of autonomous persons’ (Beauchamp, 2003). Autonomy has intrinsic value but is also instrumentally valuable to choice (Varelius, 2006). In medical ethics, the value of choice has a clear instrumental connection to autonomy, personhood, and equality (Walker, 2022). That is, choice is a realisation of one’s autonomy, and the opportunity to choose expresses that one is seen as an equal in society (Walker, 2022). In this way, autonomy incorporates choice by capturing both the ‘right to decide’ and ‘freedom of whether to decide’ (Burr & Morley, 2019).

Further differentiating autonomy from choice, which is often framed in health contexts as something that clients are ‘offered’ or ‘allowed to make’ and thus suggesting that one’s choice is dependent on another’s, autonomy concerns one’s control, agency, and competency (Entwistle et al., 2010). Callaghan et al. (2013) argue that ‘no conceptualisation of autonomy suggests that persons should be ‘abandoned’ to their own decisions without consideration of what is necessary to make genuinely autonomous decisions’. An overemphasis on choice fails to acknowledge potential limitations on autonomy that some clients have and may lead to risky choices (e.g., facilitating assisted suicide of someone who lacks the capacity to make autonomous decisions). In contrast, an overemphasis on autonomy inherently maximises respect for client capacity. In this way, too much autonomy is not inherently risky, except when it is confused with the maximisation of choice.

In a landmark publication, VSD pioneers listed autonomy among thirteen values (with ethical import) that are often implicated in system design (Friedman et al., 2013). Following VSD, autonomy is defined as the ability among those who are self-determining ‘to decide, plan, and act in ways that they believe will help them to achieve their goals and promote their values’, and its importance is expressed as fundamental to human flourishing and self-development (Friedman, 1996) (*Norm 12: Support self-directedness–goal achievement and promote values*). A clear tension between autonomy and choice is noted in VSD theory, highlighting that the maximisation of choice (or control) in the design and use of digital technology does not adequately account for and may interfere with autonomy (Friedman, 1996). Consider a non-technical user of a mood-tracking mobile application whose goal is to answer surveys to record mood states, the ability to control minute program operational details (e.g., access to modify program functions) is not necessary to achieve that goal. Recording mood states efficiently will enhance autonomy for this user, but excessive choice may interfere with autonomy by obstructing their primary goal. The VSD conceptualisation of autonomy is aligned with biomedical ethics, likewise observing that it is essential to understand that ‘autonomy is protected when users are given control over the right things at the right time’ (Friedman, 1996). Like care, in technology design, ensuring autonomy requires creating a balance: ‘machines should be designed not only to promote human autonomy but also to constrain the abdication of too much decision-making power’ (Umbrello & van de Poel, 2021).

DHTs can support autonomy by virtue of use, incorporating specific design elements, or designing for user optionality.To follow are some examples of DHTs supporting autonomy. 

• By virtue of use–Using an in-home nocturnal seizure detector supports autonomy by enabling someone to sleep in their own bed (van Andel et al., 2015)

• Incorporating specific design elements–Sending laboratory results and appropriate information to interpret the results and make informed care decisions to a patient rather than having a healthcare professional screen results (van Velsen & Grünloh, 2024)

• Designing for user optionality–Including different privacy options enabling a user to decide who has access to their health data (Mueller & Heger, 2018) 

Yet, DHTs can also harm autonomy by, for example, ‘persuading users to do something they do not want to’, such as being more physically active to inspire autonomy, without consideration for specific user goals and values relating to autonomy (Detweiler & Hindriks, 2012) (*Norm 12: Support self-directedness–goal achievement and promote values; Norm 13: Avoid persuasion, pressure, and digital addiction*). Additionally, value conflicts are evident between autonomy versus privacy and responsibility. For instance, a remote continuous health monitoring device may support autonomy by enabling someone to enjoy in-home care rather than residential care. However, continuous monitoring requires some loss of privacy, and remote digital monitoring complicates which party is held responsible for the device, data collected, and resulting care that incorporates the data (van Andel et al., 2015). Privacy versus autonomy is a recurring theme in the DHT literature, which often frames autonomy as at risk if privacy is not protected (Bolt et al., 2023) (*Norm 14: Protect personal data privacy*). In the pursuit of increasing autonomy for healthcare clients via greater control over health data, the transfer of health data online is accompanied by concerns about data security (who is handling, accessing, and securing the data), trust (whether the data is reliable), and ownership (who owns the data) (Timmermans et al., 2011). 

Two reviews of studies addressing DMHTs, by Sanches et al. (2019) and Thieme et al. (2020), identified autonomy as the most commonly addressed ethical principle in the included literature. The reviews identified that autonomy primarily focuses on respect for the voice of those with mental health concerns and their data privacy (Sanches et al., 2019), and ‘the application of privacy protecting measures to respect, and ensure confidential treatment of, peoples’ personal information’ (Thieme et al., 2020) (*Norm 14*). However, autonomy concerns more than privacy. In a discussion about the role of chatbots in mental healthcare, Vilaza and McCashin (2021) indicate that understanding value-driven external (e.g., culture) and internal (e.g., personality) factors is key to supporting the development of necessary mutual autonomy between the care recipient and caregiver, namely mutual respect, empathy, and trust. Similarly, Rughiniş et al. (2015) suggest that autonomy is enhanced by advancing transparency, truthfulness, and honesty in the design of DMHTs. Furthermore, the authors note the importance of other interconnected values in relation to advancing autonomy with DMHTs, including control, personalisation, reliability, goal-pursuit, and all other relevant moral values promoted by the intended purpose of the DMHT (Rughiniş et al., 2015). Hoffman et al. (2020) expand these autonomy-related value considerations in a study of mental health apps, drawing a clear relationship between autonomy and all personal values when advising that DMHTs should be matched to a user’s skills, goals, and personal values to ensure autonomy. Furthermore, as essential steps to strengthening autonomy with DMHTs, users should be supported in learning to identify the benefits and availability of DMHTs, make informed decisions when selecting DMHTs, and trust DMHT use in care (Hoffman et al., 2020) (*Norm 15: Educate benefits and support informed decisions*).

Young people value autonomy. They aspire to be independent, create an individual identity, and make unrestrained choices (van Dijk et al., 2023). In youth mental health, autonomy is described as a fundamental right relating to respect for personhood, responsibility, self-governance, informed decision-making, and independence (Ilyas et al., 2020). Autonomy is an important theme in the context of youth digital mental health. Like much of the literature, privacy and self-directedness are often reported as instrumental to autonomy, and thus, ensuring privacy and self-directedness in the design of DMHTs for young people is critical (Hugh-Jones et al., 2022; Kahl et al., 2020) (*Norm 12; Norm 14*). Several studies reveal numerous essential facets of autonomy in this space. A study by Alvarez-Jimenez et al. (2020) evaluating a youth digital mental health service found that young people’s sense of autonomy diminished when their access to particular integrated digital components (in this case, social media) was limited. Contrastingly, among those with unlimited access to all features, a higher sense of autonomy was reported, regardless of whether they used the added features (Alvarez-Jimenez et al., 2020). In a study about young people’s mental healthcare treatment engagement in the Netherlands, van Dijk et al. (2023) identify several ‘autonomy-supportive strategies’ in this space, including communicating the rationale of treatment options, offering multiple choices, treating young people as equals, minimising pressure (e.g., when seeking information), and acknowledging young peoples’ perspectives (e.g., emotions) (*Norm 12: Support self-directedness–choice; Norm 13*). A study by Kornfield et al. (2023) addressing a mobile phone text messaging intervention to support young people in mental healthcare reported that young people valued being able to discontinue treatment strategies delivered via DMHTs, emphasising the importance of incorporating features and user experiences that enable choice by design (*Norm 12: Support self-directedness–choice*). DMHTs for young people also pose some risk of negatively impacting autonomy. That is, DMHTs may inherently increase digital addiction and manipulation (thus reducing autonomy) (Wies et al., 2021), which significantly affects youth due to the prevalence and severity of digital addiction among younger populations (Ding & Li, 2023) (*Norm 13*).

**Fig. 4:**
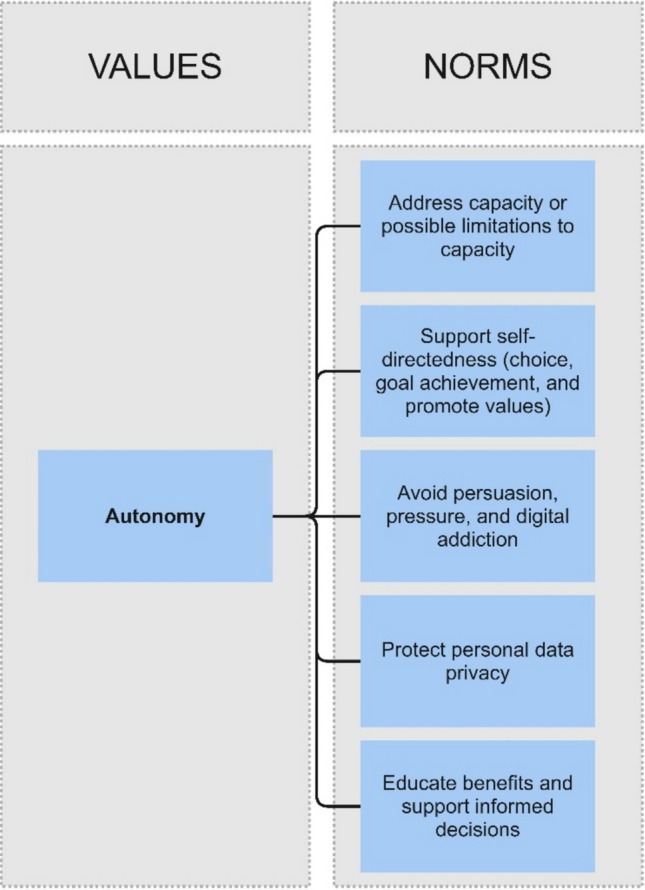
Value of autonomy component of the Framework

Figure 4 maps the norms arising from the conceptualisation of the value of autonomy above. Supplementary Fig. 1 combines the complete Framework with all components: personalisation, empowerment, and autonomy (for a document format of the Framework for proactive design, see: Supplementary Table 1).

Table 1 summarises additional practical considerations relating to each key value identified as a set of guidelines for applying the Framework. These include value synonyms, value tensions, ‘too much’ traps (i.e., risks of prioritising one value to the point of causing harm), associated values, and disvalues (van de Poel, 2020). The guidelines complement the Framework, further translating complex, multifaceted conceptualisations into basic guidelines for practitioners to consider in DMHT design processes.

**Table 1:** Guidelines for applying the Framework

	Personalisation	Empowerment	Autonomy
Synonyms	Individualisation, customisation	Self-determination, ownership	Choice, dignity of risk, independence
Tensions	Personalisation versus community-focused	Empowerment versus universal usability	• Autonomy versus privacy • Autonomy versus responsibility
‘Too much’ traps	Too much personalisation risks creating echo chambers and bias	• Too much empowerment risks removing professional expertise and efficiency • Too much empowerment risks overreliance and obligation/responsibility on clients taking their health measurements and managing their health data	Too much autonomy is only of concern when confused with too much choice: • Too much choice risks dangerous choices or choice paralysis • Too much choice risks obstructing key goals
Associated values	Self-management, responsibility, solidarity, safety, transparency, objectivity, serendipity, privacy, trust	Participation, justice, respect, autonomy, individual responsibility, ownership, independence, control, self-drive	Control, agency, competency
Disvalues	Conformity	Imposed control, obligation	Persuasion

## Application of the Framework to DMHTs

### Proactive ethical design

**Fig. 5:**
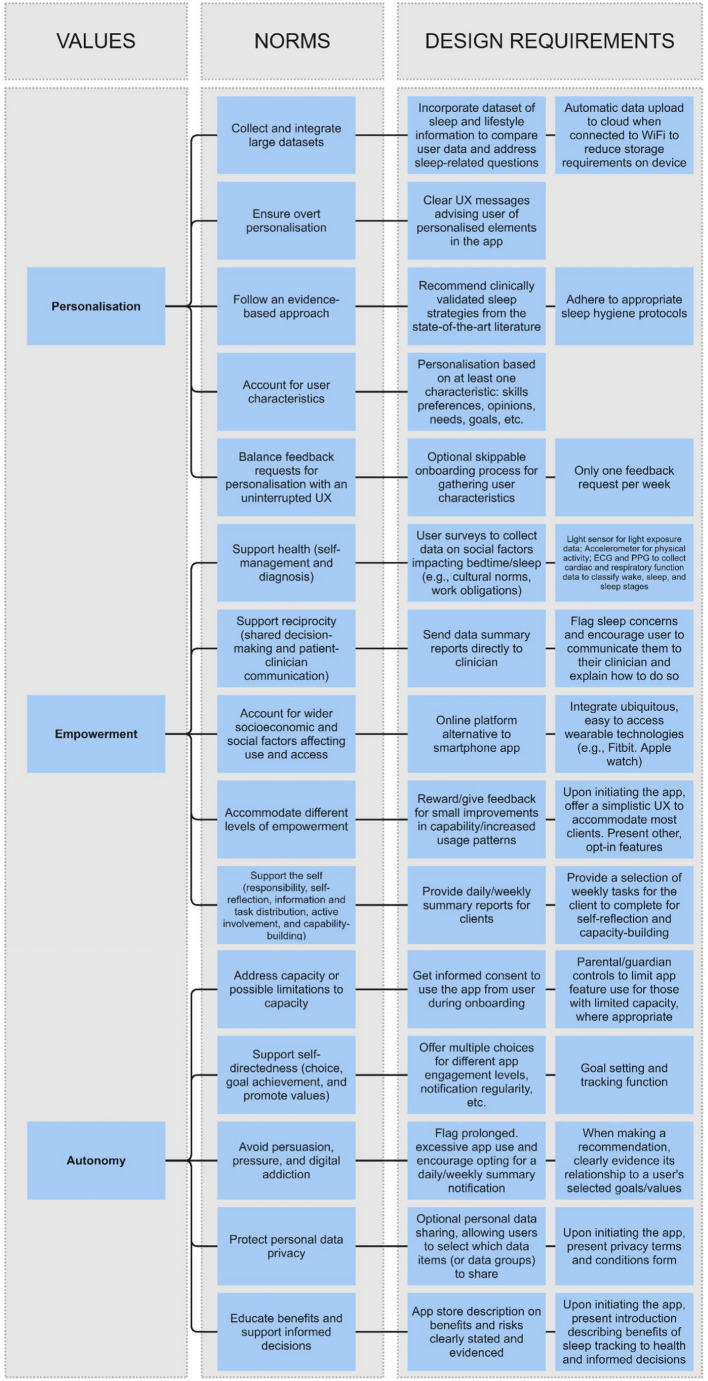

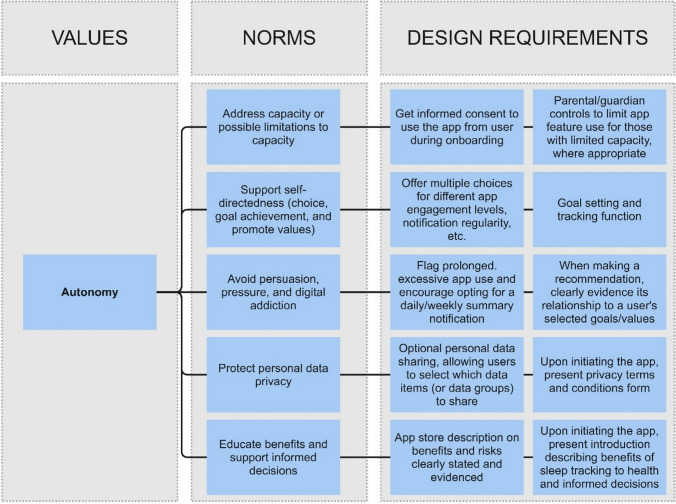
Non-comprehensive values hierarchy for the design of a mobile app and integrated wearable device for sleep tracking. The illustrative design requirements are based on various literature sources, see: Aji et al. (2019), Hicks et al. (2019), Liu et al. (2015), Walch et al. (2016), and de Zambotti et al. (2020)

**Fig. 6:**
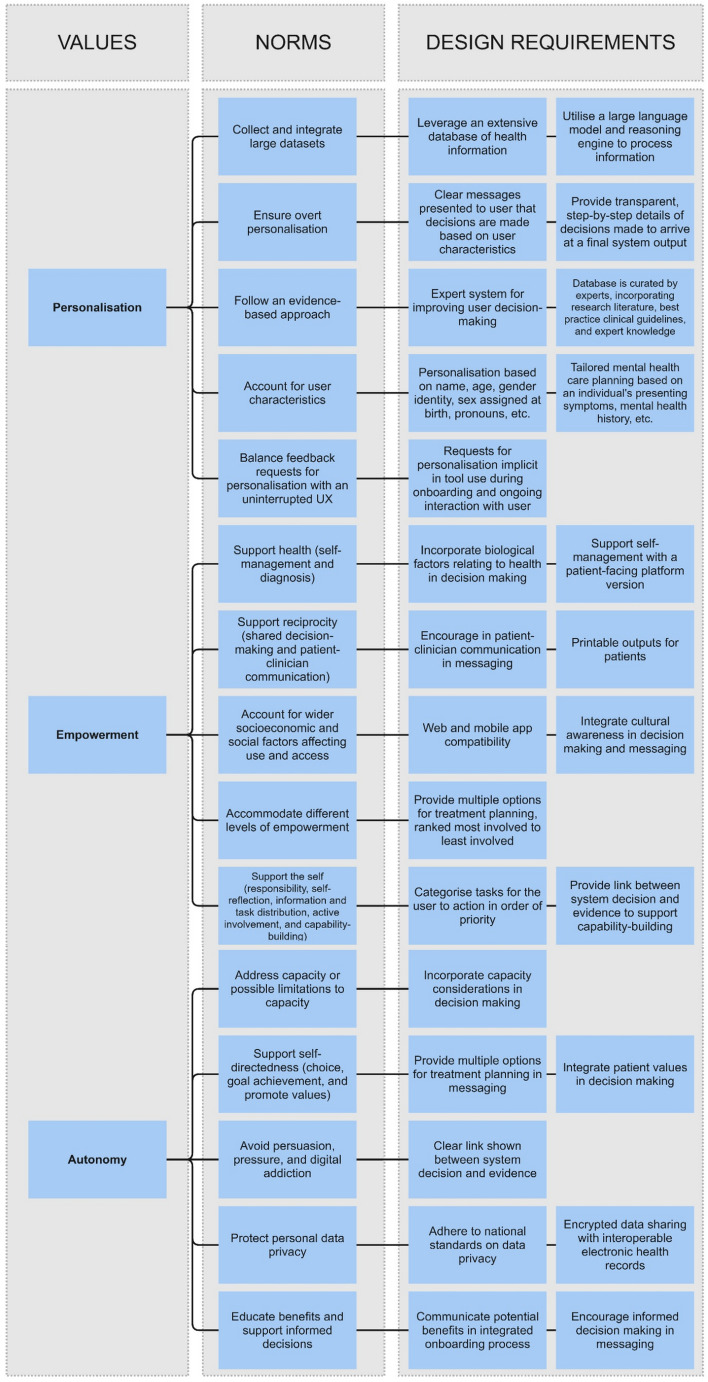

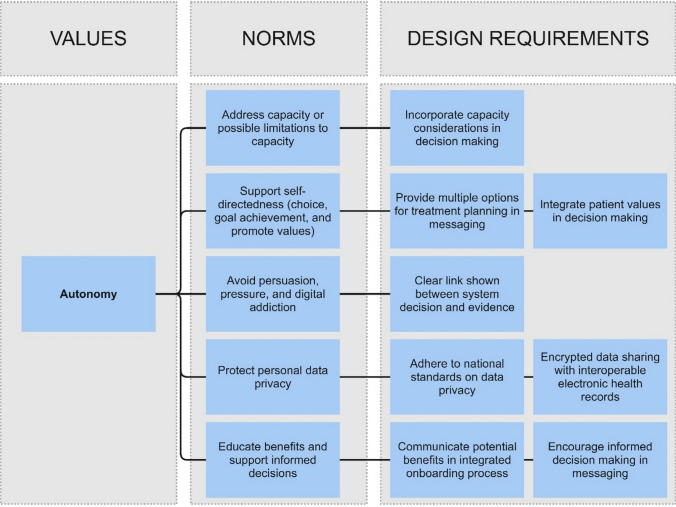
Non-comprehensive values hierarchy for the design of an AI chatbot for personalised mental healthcare guidance. The illustrative design requirements are based on an ongoing research project, see: Poulsen et al. (2025)

Following VSD, designing digital solutions depends on understanding the socio-technical context in which tools are deployed and the specific digital technology. The prototype conceptual Framework for the ethical design and evaluation of personalised youth DMHT itself serves to frame the socio-technical context, whereas the following value hierarchies illustrate its application to explicate non-comprehensive, emerging ethical design considerations using the activity of specification for two exemplary DMHTs: a mobile app and integrated wearable device for sleep tracking (Fig. 5) and a conversational generative artificial intelligence (or AI chatbot) for personalised mental healthcare guidance (Fig. 6).

Translating design norms associated with personalised youth digital mental health into illustrative design requirements requires insight into technology availability and the socio-technical context. Consider the mobile app and integrated wearable device for sleep tracking as an example and the following illustrations of translating norms into illustrative design requirements (Fig. 5 provides a non-comprehensive value hierarchy for this DMHT):

- To address *Support health–self-management and diagnosis (Norm 6)*, sleep medicine indicates that light exposure and social factors affecting light exposure (e.g., cultural norms and work obligations) are key factors underlying sleep (Walch et al., 2016). As illustrative design requirements to satisfy this norm, (1) in-built smartphone light sensors could be leveraged to collect light exposure data, and (2) user surveys can be integrated into the app to collect data on social factors impacting bedtimes and sleep quality. Furthermore, research shows that sleep quality indicators include physical activity during sleep and cardiac and respiratory function (de Zambotti et al., 2020). As another design requirement, interoperable wearables can be utilised to measure physical activity via an embedded accelerometer, cardiac function via electrocardiogram (ECG), and respiratory function via photoplethysmography (PPG).
- Employing easy-to-access, commonplace wearables (e.g., Fitbit or Apple watch) fits another design norm, *Account for wider socioeconomic and social factors affecting use and access (Norm 8)* (Hicks et al., 2019).
- To address *Follow an evidence-based approach (Norm 3)*, sleep tracking app content should adhere to proper sleep hygiene protocols (Liu et al., 2015).
- To address *Protect personal data privacy (Norm 14)* and *Accommodate different levels of empowerment (Norm 9)* app features should include optional personal data sharing and rewards for small improvements in capability or increased usage patterns, respectively (Aji et al., 2019)

This hypothetical mobile app and integrated wearable device for sleep tracking is intended to be used in everyday settings among a general young adult audience. However, for example, if the target user group were those from a vulnerable community or the context was a care setting, the illustrative design requirements would differ for the different socio-technical contexts. For instance, if the app were intended for those with vision impairment, then including text size adjustment options and text-to-speech content output would appropriately account for the norm *Address capacity or possible limitations to capacity (Norm 11)*.

### Retrospective evaluation of ethical design

To demonstrate the utility of the Framework for the retrospective evaluation of DMHT ethical design, three existing DMHTs were assessed to determine whether they are designed to support personalisation, empowerment, and autonomy using the Framework’s 15 items. Each item was operationalised as a binary criterion, i.e., a score of 1 (yes) was assigned if there was evidence that the DMHT incorporated the design norm, and 0 (no) if such evidence was absent. This resulted in a total score ranging from 0–15 for each DMHT (for a document and spreadsheet format of the Framework for retrospective evaluation, see: Supplementary Table 2 and Supplementary Data 1, respectively). Evidence considered included any explicit or implied feature of the DMHT that demonstrated alignment with a given norm, including documentation (e.g., privacy policies and developer descriptions), features observable through use (e.g., options for tailoring content or prompts encouraging user choice), and published evaluations describing the DMHT’s functions. This procedure involved two co-authors (A.P. and M.K.C.) independently reviewing the three DMHTs and applying the coding scheme. Following independent scoring, the two co-authors compared their results. Where differences in scores arose, discrepancies were discussed first between the two co-authors, and if consensus could not be reached, the issue was reviewed and resolved by consensus in conversation with the remaining co-authors. This process ensured transparency and consistency in the application of the Framework and the scores, resulting in an agreed set of scores for each of the three DMHTs.

To follow is a description of the three DMHTs evaluated. First, Mello (https://www.mello.org.au/), a mental health smartphone app targeted at young people that provides tools and techniques intended to alleviate stuck thinking (i.e., rumination and worry). Second, Mentat AI (https://www.mentat-ai.com/), a mental health app incorporating evidence-based cognitive behavioural therapy techniques and real-time support provided by an AI-driven emotional agent. Third, Earkick (https://earkick.com/), an AI chatbot (accessible via a web browser or app) that aims to provide personalised, real-time support and tracking for mental health.

**Fig. 7:**
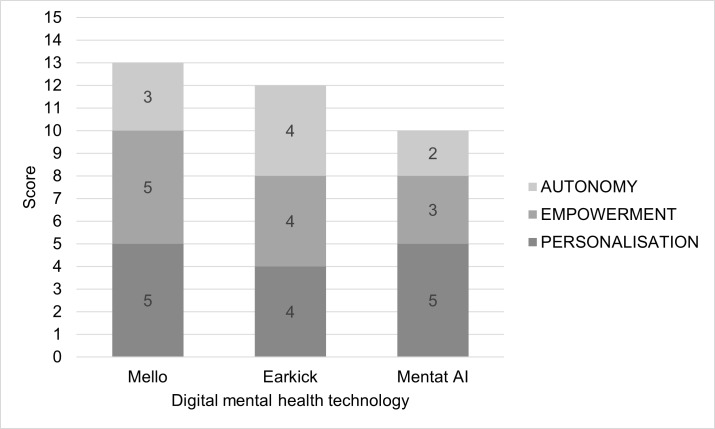
Retrospective evaluation scores for three existing DMHTs

The evaluation resulted in scoring Mello highest (13/15), followed by Earkick (12/15), and finally Mentat AI (10/15) (Fig. 7). To follow are several highlights emerging from the evaluation. Only Earkick demonstrably accounted for the norm *Address capacity or possible limitations to capacity (Norm 11)*. Specifically, Earkick includes audio and text user input modes and text-to-speech output, which may enable users with impairments that present possible limitations to capacity (e.g., vision and hearing impairment) to use Earkick effectively. Mello was the only DMHT evaluated that addressed *Support reciprocity–shared decision-making and patient-clinician communication (Norm 7)* as the app content explicitly encourages engaging in collaborative care, i.e., actively discussing insights drawn from the app with a mental health professional. Whereas Earkick and Mello did not discernibly account for the norm *Avoid persuasion, pressure, and digital addiction (Norm 13)*, Mentat AI makes it clear multiple times during the onboarding process that the user should not disregard professional medical advice or delay seeking it based on the information provided in the app. Furthermore, Mentat AI consistently asks the user if they want further details about multiple recommended activities presented for the user to action rather than assuming they want to pursue one particular recommendation. Supplementary Table 3 details the authors’ full retrospective evaluation of the three DMHTs.

## Discussion

### Framework contributions and theoretical positioning

This work contributes a VSD perspective and a subsequent prototype conceptual Framework for the ethical design and evaluation of personalised youth DMHT. Furthermore, it contributes illustrative applications of the Framework for proactive design and retrospective evaluation of DMHT. The Framework’s three core values–personalisation, empowerment, and autonomy–and the associated 15 design norms are conceptualised here as fundamentally interconnected rather than independent ethical considerations that together advance towards personalised care in this context. The conceptual investigation here elaborates on these foundational yet non-exhaustive values and norms to show that data-driven personalisation must be coupled with genuine user empowerment and autonomy to achieve ethical, personalised DMHT design. The illustrative applications highlight the Framework’s dual utility, as per the VSD approach. First, for proactively guiding ethical design considerations and developing design requirements during development (as demonstrated with the sleep tracking and AI chatbot examples). And, second, for retrospectively evaluating existing technologies for ethical alignment. The retrospective evaluation of three existing DMHTs shows variable ethical design implementation (ranging from 10–13 out of 15 design norm criteria), with common gaps in addressing capacity limitations and avoiding digital manipulation. This highlights areas where current youth DMHT design practices fall short of ethical ideals.

The prototype conceptual Framework contributes to a growing body of value-based approaches to digital technology design, each employing different methodologies to identify and address ethical considerations, resolve value tensions, and develop value-driven design requirements. While the VSD approach that frames this work begins with a conceptual investigation to derive values from domain-specific literature, alternative techniques such as speculative design scenarios and various stakeholder engagement methods offer complementary insights. For instance, Risnes et al.’s (2024) exploration of value dilemmas in brain monitoring technology through speculative scenarios demonstrates how developing and empirically exploring fictional narratives can reveal stakeholder nuances, competing priorities, and value and norm tensions that may not emerge through literature analysis alone. The authors’ identification of tensions between individual and collective benefits (Risnes et al., 2024), while contextually different from youth digital mental health, shares conceptual similarities with the personalisation versus community-focused tension identified here (see Table 1). Similarly, but again contextually distinct, Gil et al.’s (2025) research on value-based agricultural data spaces demonstrates how direct stakeholder engagement can reveal competing domain-specific value priorities (e.g., users prioritising privacy, control, and trust and technology providers emphasising autonomy and human welfare) that align with but contextualise broader ethical principles differently than literature analysis. Gil et al.’s (2025) identification of fundamental tensions between one stakeholder group’s control over data and another stakeholder group’s autonomy mirrors the tensions that can emerge around values implicated in youth digital mental health contexts. The convergence of different methodological approaches highlights that certain value tensions are shared across domains, and some are context-specific. This reinforces the need for systematic approaches to value conceptualisation and highlights that the Framework represents one method within the broader VSD landscape, requiring integration with empirical and technical investigations for comprehensive VSD.

A critical enhancement to the Framework arises from integrating established behaviour change theories, which provide empirically grounded constructs aligned with the three values and strengthen the value conceptualisation and specification processes. The three basic psychological needs within self-determination theory (SDT)—autonomy, competence, and relatedness (Ryan & Deci, 2017)—offer a theoretical foundation that enriches the Framework’s emphasis on autonomy and empowerment. Following SDT, Ryan and Deci’s (2000) conceptualisation of autonomy as the need to feel volitional and self-directed in one’s actions harmonises with the norm *Support self-directedness (Norm 12)*. Similarly, the competence construct in SDT aligns with the empowerment value, particularly with the norm *Support the self–capability-building (Norm 10)*. Additionally, integrating SDT into the Framework is positioned to provide additional granularity for specifying design requirements aligned with the value of personalisation. For example, the competence construct, which highlights skill development trajectories and growth (Ryan & Deci, 2000), further informs design requirements for the personalisation norm *Collect and integrate large datasets* (*Norm 1)* by, for instance, requiring that DMHTs capture users’ skill trajectories with longitudinal tracking and thereafter personalise content difficulty and intervention timing based on demonstrated rather than assumed capabilities. In this way, SDT integration ensures personalisation serves intrinsic psychological development rather than surface-level customisation. The behaviour change wheel framework offers another valuable lens, with Michie et al.’s (2011) COM-B model (capability, opportunity, motivation) providing additional theory to enhance appreciation of behaviour change in the Framework. For example, the COM-B model’s emphasis on capability, i.e., ‘psychological and physical capacity to engage in the activity concerned’ (Michie et al., 2011), aligns with the norm *Address capacity or possible limitations to capacity (Norm 11)*. Likewise, the model’s attention to social and physical opportunity, i.e., ‘factors that lie outside the individual that make the behaviour possible or prompt it’ (Michie et al., 2011), resonates with the norm *Account for wider socioeconomic and social factors affecting use and access* (*Norm 8)*. The COM-B model’s definition of motivation as ‘brain processes that energize and direct behaviour, not just goals and conscious decision-making’ (Michie et al., 2011) including habitual processes and emotional responding, provides theoretical grounding for the Framework’s emphasis on creating digital environments that support engagement through personalisation, empowerment, and autonomy rather than relying on persuasion, which the Framework discourages as per the norm *Avoid persuasion, pressure, and digital addiction (Norm 13)*.

The prototype conceptual Framework draws attention to critical, multifaceted tensions in youth digital mental health (see Table 1). In particular, it highlights the precarious equilibrium between technological personalisation and human agency, which becomes especially complex given young people’s developing autonomy and empowerment, as well as unique vulnerabilities, in digital spaces (Livingstone & Third, 2017; Smith & Shade, 2021). The personalisation versus community-focused tension encompasses not only the risk of creating echo chambers but also fundamental questions about how to balance and realise both individual and community-level adaptation and wellbeing outcomes with mental health interventions (Raymond et al., 2023). This is particularly salient for youth populations in which peer relationships and social belonging are crucial developmental needs (Newman et al., 2007). Similarly, the tension between autonomy and privacy involves key considerations about ownership and control in relationships implicated in digital health. Conflicts emerge when young people’s developing capacity for autonomous decision-making and their right to privacy intersect with legitimate interests in their wellbeing held by families, parents/guardians, educational institutions, or healthcare systems that might otherwise demand less autonomy and privacy for young people. While these value tensions cannot be universally resolved, the Framework makes these tensions explicit and advises structured approaches going forward for context-specific decision-making involving relevant stakeholders, including young people themselves as primary stakeholders in their mental healthcare.

### Limitations and future directions

Some limitations are noted. First, the Framework is currently a prototype developed from conceptual work, without empirical insights, testing, and validation. Consequently, its applicability and effectiveness in real-world settings remain unconfirmed. Therefore, it should be regarded as a foundational model intended to guide future research efforts, empirical evaluation, and iterative refinement within the domain. Adhering to VSD, iterative conceptual, empirical, and technical investigations are necessary to iteratively develop and validate the Framework. This might include a systematic review of the literature, participatory design research, structured scenario analysis, stakeholder mapping, cultural probes, and user studies. Second, following VSD, given that any list of values is non-exhaustive, the Framework establishes fundamental yet non-exhaustive ethical criteria for personalised youth DMHT; emerging values in any given application of the Framework should be incorporated in situ. Third, as the illustrative application of the Framework serves to guide practitioners in using the Framework rather than realising a finalised DMHT, the set of illustrative design requirements described are non-comprehensive. Upon further investigation, an indefinite number of design requirements could be drawn out to meet the norms and values. Fourth, due to the scope and ever-changing nature of values, this work does not engage with the diversity of value considerations in youth digital mental health contexts. These include values held by cultures, e.g., Indigenous persons (Shay et al., 2023); communities, e.g., LGBTIQ + (Bailey et al., 2024) and rural populations (Bowman et al., 2017); and organisations, e.g., mental health services (Pryjmachuk et al., 2024) and schools (Allen et al., 2017). However, the Framework provides a high-level conceptualisation and structure under which these values can be captured and incorporated into design artefacts. Last, as is the case with the majority of values-driven design work (van de Poel, 2013), the Framework presented here does not provide a means to resolve value conflicts, which should be resolved in situ, given the socio-technical context and DMHT being designed. Rather, as a step forward, it identifies key often-confronted value tensions and makes them explicit and transparent, laying the groundwork for engaged and proactive practitioners to adequately report value judgements and trade-offs made when designing personalised DMHTs for young people in combination with appropriate design methods, such as co-design and design probes. One possible method for resolving value conflicts worth investigating in future research would be to distinguish instrumental and intrinsic values in the Framework, introduce another hierarchy level for this distinction, and establish one regulating intrinsic value to resolve instrumental value tensions. For instance, following high-level guidelines or codes such as the Constitution of the World Health Organization to identity intrinsic values, ‘health,’ ‘quality of life,’ or ‘human wellbeing’ could be upheld as an intrinsic value above personalisation, empowerment, and autonomy conceptualised merely as instrumental values. In instances where tensions between these values arise, exploring and weighing the importance of each in supporting the intrinsic value could be considered to resolve conflicts. However, a more comprehensive VSD study that draws further insights from policy and empirical investigations with stakeholder input is required to develop, test, and validate the Framework in this way.

## Conclusion

This work presents a prototype conceptual Framework for the ethical design and evaluation of personalised youth DMHT, comprised of three underlying values as fundamental yet non-exhaustive ethical criteria: personalisation, empowerment, and autonomy. Moreover, illustrative applications of proactive design and retrospective evaluation are provided to demonstrate the Framework’s utility in practice and draw out associated ethical design considerations with real-world DMHTs. The straightforward Framework, guidelines, and illustrative applications presented here are positioned to streamline adoption among practitioners in research, industry, and policy environments, ultimately benefiting the realisation of ethical, personalised DMHTs for young people.

## Supplementary Information

Below is the link to the electronic supplementary material.

Supplementary file1 (PDF 56 KB)

Supplementary file2 (PDF 87 KB)

Supplementary Fig. 1: Complete Framework

Supplementary file2 (PDF 40 KB)

Supplementary file2 (PDF 41 KB)

## Data Availability

All data generated or analysed during this study are included in this published article and its supplementary information files.
