# Supplementary material for: Personalised care, youth mental health, and digital technology: A value sensitive design perspective and framework
Source: Ethics Inf Technol. 2025 Oct 22;27(4):61. doi: 10.1007/s10676-025-09866-x (PMC12546520; doi:10.1007/s10676-025-09866-x)
Supplement: Supplementary file 2 — Supplementary file2 (PDF 87 KB) [file 10676_2025_9866_MOESM2_ESM.pdf]

VALUES	NORMS		DESIGN ELEMENTS
	Collect and integrate large datasets	☑	App collects health-related data from user input to generate recommended activities for them to action
	Ensure overt personalisation	☑	After the onboarding process, the user is presented with a tailored summary of their responses during onboarding. Users are reassured that activities recommended by the app are 'Picked just for you'
	Follow an evidence-based approach	☑	During onboarding, the app advises that the app has undergone a 6-week clinical trial and the results (82%) to support the validity of the tool
	Account for user characteristics	☑	During onboarding, the user can input their name, age, and gender. Upon opening the app, the user is greeted by name
**PERSONALISATION**<br>**SCORE: 5 / 5**	Balance feedback requests for personalisation with an uninterrupted UX	☑	During onboarding, the user is asked to elect if they are willing to get push notifications fortnightly, not at all, or at specific times of the day
	Support health (self-management and diagnosis)	☑	App is a self-management tool by design, offering a diary function with which the user can record their feelings (using emojis) and thoughts (using open-ended questions). During onboarding, the app presents a mental health history questionnaire
	Support reciprocity (shared decision-making and patient-clinician communication)	☑	App content encourages collaborative care
	Account for wider socioeconomic and social factors affecting use and access	☑	App is free. User can elect a specific time to receive a push notification reminding them to use the app, supporting app usage patterns that suit their schedule appropriately
	Accommodate different levels of empowerment	☑	Using the app's diary function, after the user inputs their response, they can elect to save and incorporate it into their history to potentially address the response later, delete it so it is not included in their history to avoid addressing the response, or request a recommended activity to immediately address the response. A user is reassured that they can complete as many or as few activities as they like
**EMPOWERMENT**<br>**SCORE: 5 / 5**	Support the self (responsibility, self-reflection, information and task distribution, active involvement, and capability-building)	☑	App includes recommended activities with step-by-step instructions for the user to action (information and task distribution, active involvement, and capability-building). App features a history of the user's responses to questionnaires (self-reflection)
	Address capacity or possible limitations to capacity	☐	
	Support self-directedness (choice, goal achievement, and promote values)	☑	During onboarding, user can specify their mental health and app usage goals (can select multiple goals). When the app recommends an activity, the user can choose an alternative recommended activity or search the full activity library
	Avoid persuasion, pressure, and digital addiction	☐	
**AUTONOMY**	Protect personal data privacy	☑	During account sign-up process, the user can review the privacy policy that protects personal data. User can delete app account permanently
**SCORE: 3 / 5**	Educate benefits and support informed decisions	☑	During onboarding, the app presents potential benefits from app use. Expert knowledge is communicated through colloquial language
**TOTAL SCORE 13 / 15**			
**DMHT NAME Mello**			

VALUES	NORMS		DESIGN ELEMENTS
PERSONALISATION	Collect and integrate large datasets	☑	Chatboat collects health-related data from user input to generate tailored recommendedations
	Ensure overt personalisation	☑	Chatbot responds to the user by name. On the app, the chatbot personalises its responses to address specific concerns mentioned by the user in previous conversations. A 'commitment statement' sent to the user summarises specific goals inputted
	Follow an evidence-based approach	☐	
	Account for user characteristics	☑	Web browers landing page states that the chatbot 'remembers you and creates daily content and insights just for you'. Accounts for the user's name, occupation, social situation. On the app, personal details (e.g., name, gender, and age) are optional during onboarding.
	Balance feedback requests for personalisation with an uninterrupted UX	☑	After the initial interaction, the chatbot provides some recommendations based on the information received and asks if they are helpful. After that, it offers choices for future notifications like these (e.g., none or daily check-ins at specific times). When exiting, the app asks whether it was helpful and there is an option to request the app not to ask this question again.
SCORE: 4 / 5			
EMPOWERMENT	Support health (self-management and diagnosis)	☑	It aims to detect the user's mental state. Tracks user mood, mood stability, calmness, heart rate, etc. When the user inputs concerns, the app gives practical recommendations (e.g., if the user mentions feeling overwhelmed, it suggests breaking tasks into smaller, more manageable steps)
	Support reciprocity (shared decision-making and patient-clinician communication)	☐	
	Account for wider socioeconomic and social factors affecting use and access	☑	Web browser and mobile app access options. It is free on the web browser version, but incurs a cost on the app version. Easy-to-access wearable interoperability (specifically Apple Watch integration)
	Accommodate different levels of empowerment	☑	User can elect different chatbot personalities (e.g., one that strongly encourages the user to take action and make changes, one that focuses on self-reflection, and one that is friendly and less instructive). This feature allows for different types of recommendations and levels of commitment to change.
	Support the self (responsibility, self-reflection, information and task distribution, active involvement, and capability-building)	☑	Includes features for diarising thoughts (self-reflection) and establishing a routine (responsibility). Recommends activities for capability-building and active involvement. It provides a graph of the user's overall progress
SCORE: 4 / 5			
AUTONOMY	Address capacity or possible limitations to capacity	☑	Audio and text input options. Text-to-speech option with different voices. These options accomodate different communication preferences and capacities
	Support self-directedness (choice, goal achievement, and promote values)	☑	Includes features for setting goals
	Avoid persuasion, pressure, and digital addiction	☐	Persistent popup appears, suggesting that the user scan a QR code and download the mobile app version. After subscription, app directs to a commitment page, where users can only continue with the app by commiting to make change and track progress for better mental health, supporting self-directed behaviour
	Protect personal data privacy	☑	No user account registration is needed to use the chatbot. Privacy policy is available on the website. App asks for permission to track activities (e.g., on iPhone)
	Educate benefits and support informed decisions	☑	When asked, the chatbot provides evidence to support what it is saying (e.g., it confirms that research supports specific recommended activities for the user to action). The app dashboard has a 'Knowledge Hub' section that provides helpful tips related to mental health. However, the app does not explicitly provide the effectiveness of using the chatbot
SCORE: 4 / 5			
TOTAL SCORE 12 / 15			
DMHT NAME Earkick			

VALUES	NORMS		DESIGN ELEMENTS
	Collect and integrate large datasets	☑	App (and integrated chatbot) collects health-related data from user input to report summaries of user responses to questionnaires and generate recommended activities. Various data collected (e.g., ability to manage daily responsibilities, relationships, confidence to express opinions, previous professional mental health experiences, and feelings of belonging, restlessness, depression, effort, direction, meaning, and worthlessness)
	Ensure overt personalisation	☑	App explictly informs the user that their responses collected via an onboarding questionnaire will be incorporated to tailor recommendations to enhance mental wellbeing. It explicitly mentions that 'your focus plan is crafted to lead you towards reaching, as closely as possible, the maximum score'
	Follow an evidence-based approach	☑	During the onboarding process, the app states that it provides 'AI-driven insights and scientific methods'. The onboarding questionnaire evidences the mental health assessment scale that the questions are based on (e.g., MHC-SF and K10). Presents global statistics on mental health
**PERSONALISATION**	Account for user characteristics	☑	During onboarding, the user can input their name and age. When opening the app, it greets the user by name. Chatbot addresses the user by name
**SCORE: 5 / 5**	Balance feedback requests for personalisation with an uninterrupted UX	☑	Does not activate notifications until the user explicitly chooses to get notifications from the recommended daily activities feature (i.e., 'Activities for today)
	Support health (self-management and diagnosis)	☑	After onboarding, the user is presented with a report summarising their scores to the assessments taken during the onboarding questionnaire, chiefly reporting scores associated with mental wellbeing and stress and anxiety
	Support reciprocity (shared decision-making and patient-clinician communication)	☐	
	Account for wider socioeconomic and social factors affecting use and access	☐	App is not free (there is 3-day free trial). While users can elect to receive notifications, the timing and types of notification cannot be tailored to ensure they fit into their daily routine
**EMPOWERMENT**	Accommodate different levels of empowerment	☑	After the daily check-in, which involves users giving responses to questions, the app gives users the option to either save their responses to address them later or get immediate recommendations
**SCORE: 3 / 5**	Support the self (responsibility, self-reflection, information and task distribution, active involvement, and capability-building)	☑	The graph allows users to reflect on their previous responses in the journaling feature relating to their sleep, mood, emotions, and meditation (self-reflection). Users can ask the chatbot to provide step-by-step instructions on the recommended activities (task distribution). The app does not track the user's involvement with its recommendations (limited capability-building tracking).
	Address capacity or possible limitations to capacity	☐	
	Support self-directedness (choice, goal achievement, and promote values)	☐	The app only provides guided meditation. All other mental health-related recommendations are from the chatbot. The user can't specify their goals or track habits
	Avoid persuasion, pressure, and digital addiction	☑	During onboarding, the app explicitly notes that the user should not disregard professional medical advice or delay seeking it based on the app information. Chatbot regularly asks whether the user wants further details regarding the recommendations provided
**AUTONOMY**	Protect personal data privacy	☑	Privacy policy is not provided at sign-up, but users can locate the information in app
**SCORE: 2 / 5**	Educate benefits and support informed decisions	☐	
**TOTAL SCORE 10 / 15**			
**DMHT NAME Mentat AI**			
