# Supplementary figures and images for: Personalised care, youth mental health, and digital technology: A value sensitive design perspective and framework
Source: Ethics Inf Technol. 2025 Oct 22;27(4):61. doi: 10.1007/s10676-025-09866-x (PMC12546520; doi:10.1007/s10676-025-09866-x)

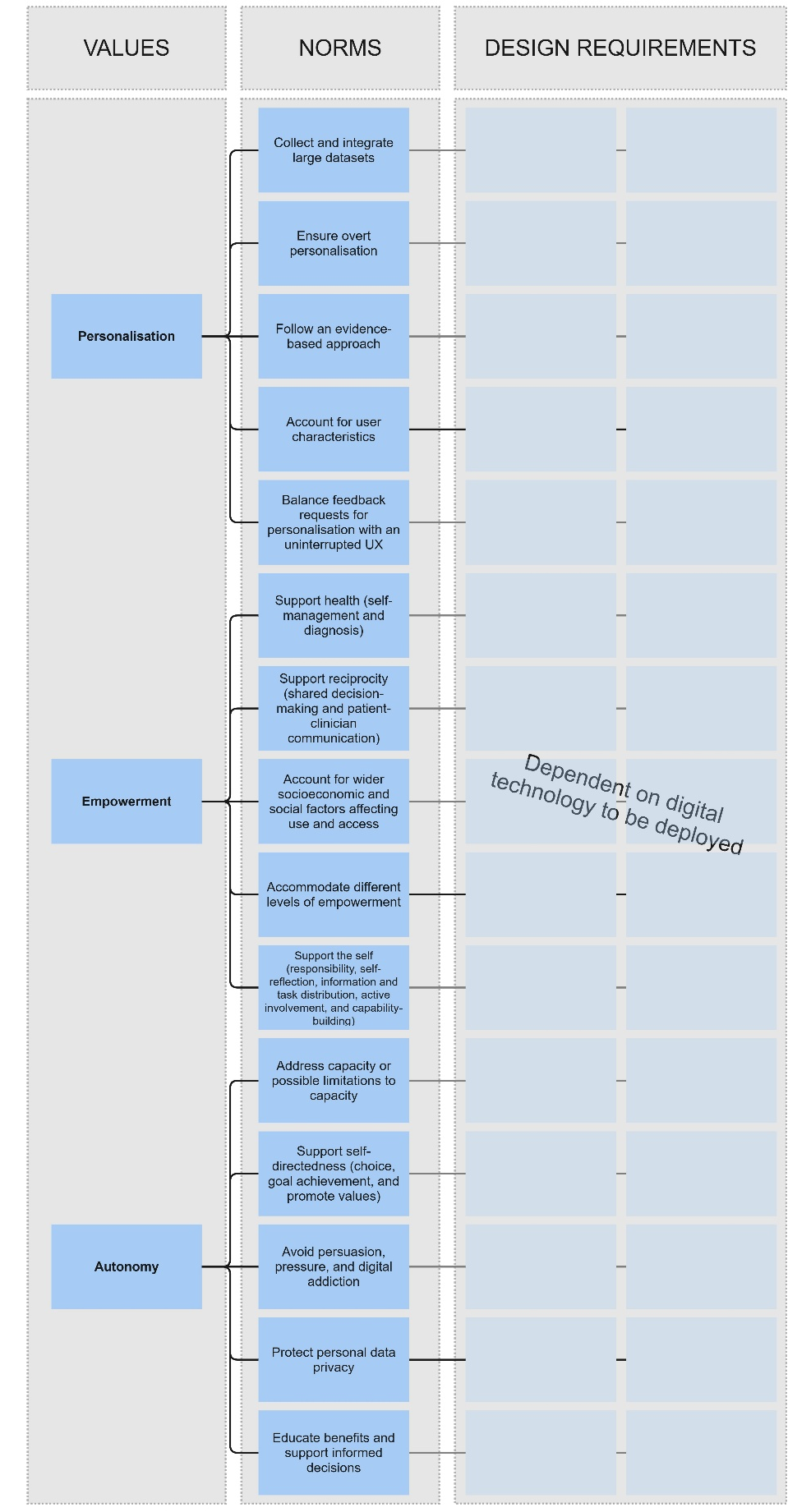

Supplement: Supplementary file 3 — Supplementary Fig. 1: Complete Framework [file 10676_2025_9866_MOESM3_ESM.png]
