# Supplementary material for: Personalised care, youth mental health, and digital technology: A value sensitive design perspective and framework
Source: Ethics Inf Technol. 2025 Oct 22;27(4):61. doi: 10.1007/s10676-025-09866-x (PMC12546520; doi:10.1007/s10676-025-09866-x)
Supplement: Supplementary file 5 — Supplementary file2 (PDF 41 KB) [file 10676_2025_9866_MOESM5_ESM.docx]

**Supplementary Table 2: Full Framework for retrospective evaluation (document format).**

| **VALUES** | **NORMS** | **DESIGN ELEMENTS**  If the DMHT has a design element that demonstrably meets a design norm, note it and tick the box. Sum ticks to calculate scores. |
| --- | --- | --- |
| **Personalisation**  **SCORE … / 5** | Collect and integrate large datasets |  |
|  | Ensure overt personalisation |  |
|  | Follow an evidence-based approach |  |
|  | Account for user characteristics |  |
|  | Balance feedback requests for personalisation with an uninterrupted UX |  |
| **Empowerment**  **SCORE … / 5** | Support health (self-management and diagnosis) |  |
|  | Support reciprocity (shared decision-making and patient-clinician communication) |  |
|  | Account for wider socioeconomic and social factors affecting use and access |  |
|  | Accommodate different levels of empowerment |  |
|  | Support the self (responsibility, self-reflection, information and task distribution, active involvement, and capability-building) |  |
| **Autonomy**  **SCORE … / 5** | Address capacity or possible limitations to capacity |  |
|  | Support self-directedness (choice, goal achievement, and promote values) |  |
|  | Avoid persuasion, pressure, and digital addiction |  |
|  | Protect personal data privacy |  |
|  | Educate benefits and support informed decisions |  |

**TOTAL SCORE: … / 15**
